# Association between central venous pressure measurement and outcomes in critically ill patients with severe coma

**DOI:** 10.1186/s40001-022-00981-9

**Published:** 2023-01-18

**Authors:** Xin Tong, Xin Feng, Chuanzhi Duan, Aihua Liu

**Affiliations:** 1 Beijing Neurosurgical Institute and Beijing Tiantan Hospital, Capital Medical University, Beijing, 100050 China; 2 Department of Cerebrovascular Surgery, Engineering Technology Research Center of Education Ministry of China On Diagnosis and Treatment of Cerebrovascular Disease, Neurosurgery Center, Zhujiang Hospital, Southern Medical University, Guangzhou, 510280 Guangdong China; 3 Guangdong Provincial Key Laboratory On Brain Function Repair and Regeneration, Guangdong, 510280 China; 4 China National Clinical Research Centre for Neurological Diseases, Beijing, 100070 China

**Keywords:** Central venous pressure, Coma, In-hospital mortality, Glasgow Coma Scale

## Abstract

**Background:**

The use of central venous pressure (CVP) measurements among (intensive care unit) ICU patients with severe coma has been questioned. This study aimed to investigate the application value of CVP in this population.

**Methods:**

Data stored in the ICU Collaborative Research Database (eICU-CRD) and Medical Information Mart for Intensive Care III (MIMIC-III) database were reviewed. Critically ill patients with a Glasgow Coma Scale (GCS) score of 3–8 were included. The primary outcome was the in-hospital mortality rate. The statistical approaches used included multivariable Cox regression, propensity score matching (PSM), inverse probability treatment weighting (IPTW), stabilized IPTW, and restricted cubic splines (RCS) to ensure the robustness of our findings.

**Results:**

In total, 7386 patients were included in the study. Early CVP measurement was independently associated with in-hospital mortality [hazard ratio, 0.63; *p* < 0.001] in patients with severe-to-moderate coma. This result was robust in the PSM, sIPTW, and IPTW cohorts. For all patients with CVP measurements, the RCS curves showed that the risk of in-hospital mortality increased as the initial CVP time was delayed. In addition, early CVP measurement was significantly associated with lower ICU mortality, 28-day mortality, and 365-day mortality and a significantly higher number of ventilator-free days.

**Conclusion:**

Early CVP measurement could improve clinical outcomes in critically ill patients with severe coma

**Supplementary Information:**

The online version contains supplementary material available at 10.1186/s40001-022-00981-9.

## Background

Central venous pressure (CVP) is the pressure recorded in the superior vena cava or the right atrium and, to a lesser extent, the left ventricular preload [1], reflecting venous return and right ventricular function [2]. Thus, CVP measurements could help in fluid management [3]. However, the CVP can also be influenced by thoracic, pericardial and abdominal pressures, complicating its understanding. Recently, several studies have found that the CVP cannot predict fluid responsiveness and only higher or lower CVP values may have negative or positive predictive values [4–6]. Therefore, the application of CVP measurement has been questioned. Some researchers have suggested that although there could be some limitations to using the CVP to assist fluid resuscitation, it would be better to fully understand and address these limitations, rather than abandoning them completely [3, 7]. The CVP levels still provide significant information about patient's cardiocirculatory status.

Many clinical studies have reported that higher CVP values may be associated with patients’ poor outcomes in different conditions, such as patients undergoing cardiac surgery [11], early Fontan failure [10], acute kidney injury (AKI) [8, 9], patients undergoing cardiopulmonary bypass surgery [9], and critically ill patients [2]. A controlled lower CVP can also reduce blood loss during hepatectomy [12]. Meanwhile, CVP measurement has been reported to shorten the time to be medically fit for discharge and benefit the clinical outcomes for patients with acute respiratory distress syndrome or sepsis [13–15]. Some researchers have suggested using the CVP as a stopping rule for fluid infusion [3, 7]. However, to our knowledge, the value of CVP measurements in patients with coma remains unclear. Fluid administration could increase cardiac output; however, it could also increase the risk of hydrostatic pressure or edema formation. Theoretically, this situation could be more complicated in critically ill comatose patients, and CVP may be beneficial in these conditions.

Therefore, the purposes of this study were to investigate 1) the association between early CVP measurement and patients’ outcomes and 2) the association between initial CVP time and all-cause mortality of intensive care unit (ICU) patients with severe coma.

## Methods

### Study design and population

Data were collected from patients in the ICU Collaborative Research Database (eICU-CRD) [16] and the Medical Information Mart for Intensive Care III (MIMIC-III) [17]. Patient characteristics available in both databases included demographic information, vital signs, laboratory test results, diagnoses, medical histories, treatments and so on. Detailed information on these two databases can be found in PhysioNet [18].

Patients with severe coma were identified using the Glasgow Coma Scale (GCS). The GCS was first published in 1975 and is the most widely applied outcome measure in clinical settings and researches on brain injury [19, 20]. Currently, the GCS is recommended by lots of countries to use as a measurement for primary outcome. A severe coma is defined as a score of 3–8 [21]. We only collected the GCS scores before sedative drug use on the first day for each patient. For patients with multi-ICU admissions, we only include the first admission. Patients aged < 18 years or ≥ 90 years were excluded. An early CVP measurement was defined as a CVP measurement in the first 24 h after first ICU admission. According to early CVP, the entire cohort was divided into CVP and non-CVP groups. The initial CVP time and CVP values were also collected. All comorbidities were identified based on recorded ICD-9 or APACHE components. We only collected the first value within 24 h of ICU admission for vital signs and laboratory test results.

In clinical practice, there may be many reasons for coma development. Due to the nature of retrospective research, we cannot accurately determine the direct cause of coma in patients. Therefore, we collected the primary diagnoses of patients and classified them according to ICD-9 for different diseases, such as circulatory disease and injury disease.

### Study outcome

All-cause in-hospital mortality rate was set as the primary outcome. The secondary outcomes included 28-day mortality; 365-day mortality; ICU mortality; MV-free days; vasopressor-free days within 28 days after ICU admission; intravenous fluid (IVF) volumes (ml) of the first, second, and third days after ICU admission; and incidences of AKI within 2 days and 7 days after ICU admission [22].

### Statistics analysis

Continuous variables are illustrated as mean ± standard deviation (SD). Categorical variables are presented as total number and percentage. The Chi-square test was applied to compare proportions, and the T-test or Wilcoxon rank-sum test was used for continuous variables. Early CVP was analyzed as a categorical variable for the primary analysis in the whole cohort, and the CVP value was analyzed as a continuous variable in the CVP group. Multivariable Cox regression was used for all outcomes to adjust for confounders that may affect the outcomes. Variables with a *P* value of < 0.2 in the univariable analysis were enrolled in the multivariable analysis.

We used several sensitivity analyses to ensure the consistency of the findings, including propensity score matching (PSM), inverse probability treatment weighting (IPTW), and stabilized IPTW (sIPTW). Patients’ propensity score was assessed using a multivariable logistic analysis to minimize the covariate imbalance between the CVP and non-CVP groups. To compare secondary outcomes, the standardized mean differences and statistical significance of parameters between the CVP and non-CVP groups in the PSM cohort were calculated using the Chi-square test or t-test. We also used restricted cubic spline (RCS) curves based on multivariable Cox regression to evaluate the relationship between the initial CVP time or value and the primary outcome. All statistical analyses were performed using R software.

## Results

### Baseline information

**Fig. 1:**
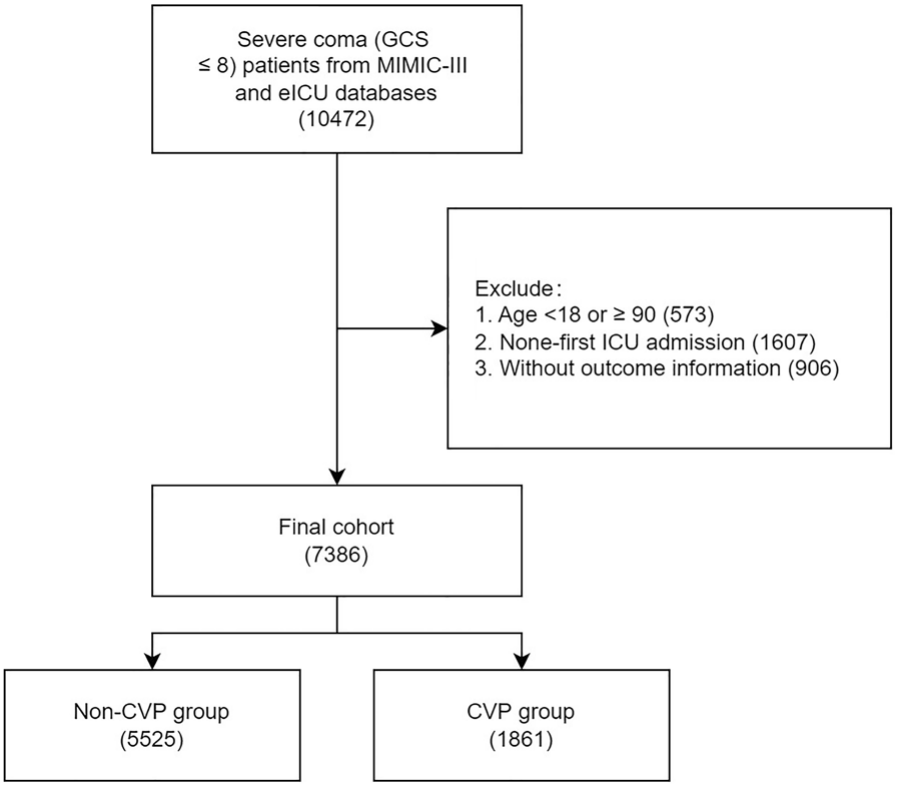
Flowchart of patients’ selection

A total of 26,2391 ICU admissions were reviewed from the eICU-CRD and MIMIC-III databases. After exclusion, 7386 patients were included in the final analysis, including 1861 patients with CVP and 5525 patients without CVP (Fig. 1). The mean GCS was 5.46 ± 1.99. The mean age of the entire cohort was 62.82 ± 16.70 years, and 3170 (42.9%) patients were female. The total in-hospital and ICU mortality rates were 41.0% (3031/7386) and 33.2% (2455/7386), respectively. Patients in the CVP group had more comorbidities, larger CCI, higher SOFA scores, lower GCS scores, lower vital signs, and greater use of sedative drugs, vasopressor drugs, and MV than those in the non-CVP group. The details of the original and PSM cohorts are presented in Table 1. In the CVP group, the initial CVP time was 5.21 ± 5.79 h and mean initial CVP value was 12.15 ± 8.79 cmH2O. Information on the missing values is demonstrated in Additional file 1: Table S1.

**Table 1:** Baseline information of original cohort and propensity scored matching cohort

	Non-CVP (*n* = 5528)	CVP (*n* = 1861)	*P* value	Non-CVP (*n* = 1241)	CVP (*n* = 1241)	*P* value
Age (mean (SD))	62.52 (17.22)	63.72 (15.02)	0.074	61.94 (16.47)	63.20 (15.25)	0.08
Gender (%)			0.109			0.015
Male	3079 (55.7)	1137 (61.1)		744 (60.0)	753 (60.7)	
Female	2446(44.3)	724 (38.9)		497 (40.0)	488 (39.3)	
GCS (mean (SD))	5.74 (1.92)	4.62 (1.97)	0.575	5.13 (1.98)	4.74 (2.00)	0.2
Ethnicity (%)			0.221			0.083
White	3892 (71.1)	1343 (72.4)		906 (73.0)	902 (72.7)	
Black	799 (14.6)	173 (9.3)		141 (11.4)	119 (9.6)	
Asian	107 (2.0)	37 (2.0)		28 (2.3)	28 (2.3)	
Hispanic	206 (3.8)	51 (2.8)		40 (3.2)	40 (3.2)	
Other	470 (8.6)	250 (13.5)		126 (10.2)	152 (12.2)	
Unit (%)			0.738			0.321
CICU	946 (17.1)	913 (49.1)		359 (28.9)	538 (43.4)	
MICU	1227 (22.2)	307 (16.5)		291 (23.4)	264 (21.3)	
MSICU	1978 (35.8)	395 (21.2)		385 (31.0)	264 (21.3)	
NICU	451 (8.2)	54 (2.9)		33 (2.7)	30 (2.4)	
SICU	923 (16.7)	192 (10.3)		173 (13.9)	145 (11.7)	
Primary diagnosis (%)			0.465			0.191
Circulatory disease	2160 (39.1)	1049 (56.4)		529 (42.6)	630 (50.8)	
Digestive disease	179 (3.2)	74 (4.0)		60 (4.8)	54 (4.4)	
Endocrine disease	186 (3.4)	50 (2.7)		42 (3.4)	31 (2.5)	
Infectious disease	391 (7.1)	175 (9.4)		146 (11.8)	155 (12.5)	
Injury disease	733 (13.3)	154 (8.3)		160 (12.9)	119 (9.6)	
Mental disease	61 (1.1)	11 (0.6)		10 (0.8)	8 (0.6)	
Neoplasm disease	157 (2.8)	41 (2.2)		30 (2.4)	29 (2.3)	
Nervous disease	422 (7.6)	45 (2.4)		34 (2.7)	28 (2.3)	
Respiratory disease	743 (13.4)	126 (6.8)		118 (9.5)	97 (7.8)	
Urinary disease	69 (1.2)	10 (0.5)		8 (0.6)	6 (0.5)	
Other disease	424 (7.7)	126 (6.8)		104 (8.4)	84 (6.8)	
CCI (mean (SD))	4.00 (2.46)	4.51 (2.41)	0.209	4.23 (2.67)	4.53 (2.50)	0.114
CHF (%)	653 (11.8)	384 (20.6)	0.241	200 (16.1)	244 (19.7)	0.093
AF (%)	686 (12.4)	450 (24.2)	0.308	207 (16.7)	295 (23.8)	0.177
Renal (%)	514 (9.3)	199 (10.7)	0.046	140 (11.3)	143 (11.5)	0.008
Liver (%)	181 (3.3)	82 (4.4)	0.059	75 (6.0)	70 (5.6)	0.017
COPD (%)	416 (7.5)	195 (10.5)	0.103	122 (9.8)	144 (11.6)	0.057
CAD (%)	315 (5.7)	533 (28.6)	0.639	128 (10.3)	310 (25.0)	0.392
Stroke (%)	1018 (18.4)	168 (9.0)	0.276	108 (8.7)	100 (8.1)	0.023
MT (%)	436 (7.9)	158 (8.5)	0.022	116 (9.3)	116 (9.3)	< 0.001
HR (mean (SD))	92.80 (24.26)	91.30 (21.60)	0.065	94.68 (24.81)	93.07 (22.76)	0.067
MAP (mean (SD))	87.32 (25.65)	80.59 (21.41)	0.285	81.80 (22.41)	80.37 (21.40)	0.065
Temperature (mean (SD))	36.68 (4.12)	36.24 (2.66)	0.128	36.30 (2.97)	36.26 (2.46)	0.016
WBC (mean (SD))	13.61 (12.62)	14.56 (11.25)	0.079	14.55 (14.76)	15.13 (12.44)	0.043
Hemoglobin (mean (SD))	11.84 (2.51)	10.90 (2.52)	0.373	11.31 (2.63)	10.95 (2.57)	0.14
Platelet (mean (SD))	223.01 (105.97)	199.98 (100.87)	0.223	211.05 (104.75)	203.36 (104.50)	0.074
Sodium (mean (SD))	138.98 (6.56)	137.96 (5.71)	0.166	138.85 (6.76)	138.09 (5.83)	0.12
Potassium (mean (SD))	4.18 (0.90)	4.35 (0.93)	0.192	4.28 (1.00)	4.37 (0.94)	0.094
Bicarbonate (mean (SD))	22.80 (5.85)	22.10 (5.11)	0.128	21.36 (6.02)	21.61 (5.44)	0.043
Chloride (mean (SD))	103.58 (7.70)	105.68 (7.12)	0.283	104.52 (7.90)	105.54 (7.39)	0.133
BUN (mean (SD))	28.15 (23.83)	27.21 (21.89)	0.041	30.31 (23.69)	28.69 (22.48)	0.07
Lactate (mean (SD))	4.33 (4.23)	3.85 (3.38)	0.128	4.22 (3.76)	3.81 (3.37)	0.115
Sedative (%)	2113 (38.2)	1387 (74.5)	0.786	792 (63.8)	923 (74.4)	0.23
Vasopressor (%)	1492 (27.0)	1229 (66.0)	0.85	663 (53.4)	811 (65.4)	0.245
MV (%)	4000 (72.4)	1584 (85.1)	0.315	1033 (83.2)	1071 (86.3)	0.085
SOFA (mean (SD))	7.35 (3.39)	9.25 (3.51)	0.549	9.40 (3.52)	9.68 (3.63)	0.08
Database (%)			0.622			0.492
MIMIC-IV	1726 (31.2)	1132 (60.8)		450 (36.3)	746 (60.1)	
eICU	3799 (68.8)	729 (39.2)		791 (63.7)	495 (39.9)	

*CVP* central venous pressure, *GCS* Glasgow Coma Scale, *CCI* Charlson Comorbidity Index, *TBI* traumatic brain injuries, *CHF* congestive heart failure, *AF* atrial fibrillation, *COPD* chronic obstructive pulmonary disease, *CAD* coronary artery disease, *HR* heart rate, *MAP* mean arterial pressure, *WBC* white blood cell, *BUN* blood urea nitrogen, *MV* mechanical ventilation, *SOFA* Sequential Organ Failure Assessment

### Primary outcome and sensitivity analysis

CVP was conducted in 18.7% of the non-surviving patients and 29.7% of the surviving patients. After adjustment for primary diagnosis, age, sex, GCS, ethnicity, unit type, AF, malignant tumor, renal disease, stroke, liver disease, CHF, CAD, COPD, MAP, temperature, heart rate, lactate, hemoglobin, platelet count, bicarbonate, BUN, WBC count, potassium, chloride, sedative use, vasopressor use, MV use, SOFA score, CCI, and database type, early CVP was significantly associated with lower all-cause in-hospital mortality in the whole cohort (hazard ratio [HR] 0.63; 95% confidence intervals [CI] 0.55–0.71; *P* < 0.001), and remained robust in the PSM cohort (HR, 0.58; 95% CI 0.51–0.66; *P* < 0.001), IPTW cohort (HR 0.65; 95% CI 0.56–0.75; *P* < 0.001), and sIPTW cohort (HR 0.64; 95% CI 0.55–0.74; *P* < 0.001) (Table 2). The full results of the different multivariate models are shown in Additional file 1: Tables S2-S5.

**Table 2:** Primary outcome analysis with different methods

Method	Hazard ratio	CI		*P* value
		2.5%	97.5%	
Original cohort	0.63	0.55	0.71	< 0.001
PSM cohort	0.58	0.51	0.66	< 0.001
IPTW cohort	0.65	0.56	0.75	< 0.001
sIPTW cohort	0.64	0.55	0.74	< 0.001

*PSM* propensity scored matching, *IPTW* inverse probability treatment weighing, *sIPTW* inverse probability treatment weighing

**Fig. 2:**
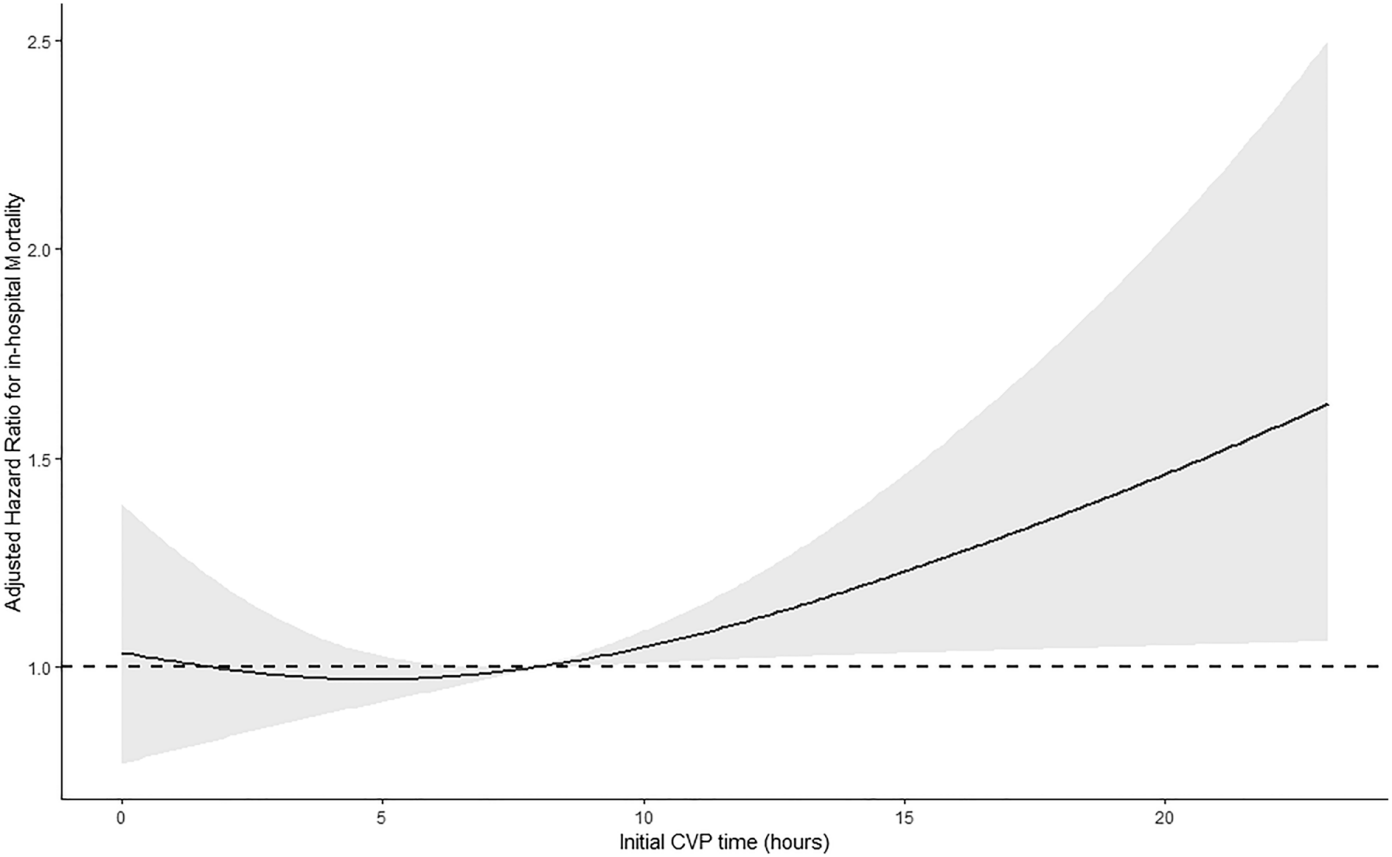
Restricted cubic spline (RCS) curves of associations between initial central venous pressure (CVP) time and all-cause in-hospital mortality. Results were adjusted for primary diagnoses, Glasgow Coma scale, unit, congestive heart failure, atrial fibrillation, coronary artery disease, stroke, heart rate, temperature, white blood cell count, hemoglobin, sodium, bicarbonate, chloride, blood urea nitrogen, lactate, sedative use, vasopressor use, mechanical ventilation use, SOFA scale, and days in ICU. RCS regression models were conducted with 3 knots at the 10th, 50th, and 90th percentiles of initial CVP time. The red lines represent the 95% confidence intervals for the spline model

The initial CVP time (hours) and value (cmH2O) after ICU admission were both associated with in-hospital mortality in the CVP group, with HRs of 1.02 (95% CI 1.01–1.03; *P* = 0.034) and 1.02 (95% CI 1.01–1.02; *P* < 0.001), respectively. In general, the RCS curve shows that with the delay in the initial CVP time, the risk of all-cause mortality in hospital increases (Fig. 2).

### Secondary outcomes

Compared with patients without CVP measurement, those with CVP have a lower ICU mortality (44.4% vs. 27.8%; *P* < 0.001), lower 28-day mortality (in MIMIC-III only, 37.8% vs. 20.6%; *P* < 0.001), lower 365-day mortality (in MIMIC-III only, 50.9% vs. 30.8%; *P* < 0.001), and a more significant number of vasopressor-free days (in MIMIC-III only, 16.95 ± 13.31 days vs. 21.15 ± 11.00 days; *P* < 0.001) (Table 3). However, patients in the CVP group as have a higher 2-day (69.5% vs. 75.5%, *P* = 0.001) and a higher 7-day (77.3% vs. 81.2%, *P* = 0.018) AKI rates. Moreover, no association was found between the number of MV-free days (20.79 ± 9.86 vs. 21.22 ± 9.80; *P* = 0.272). Compared to patient without CVP, patients in the CVP group have a higher IVF volume on day 1 (3100.88 ± 3163.26 ml vs. 4167.19 ± 3838.39 ml; *P* < 0.001) and lower IVF volumes on day 2 (2274.16 ± 2405.90 ml vs. 2058.66 ± 2357.80 ml; *P* = 0.045) and day 3 (2178.88 ± 2492.15 ml vs. 1647.62 ± 2097.16 ml; *P* < 0.001).

**Table 3:** Secondary outcome analysis with sIPTW cohort

	non-CVP	CVP	*P* value
Primary outcome			
Hospital mortality	628 (50.6)	422 (34.0)	< 0.001
Secondary outcomes			
ICU mortality	551 (44.4)	345 (27.8)	< 0.001
28-day mortality*	170 (37.8)	154 (20.6)	< 0.001
365-day mortality*	229 (50.9)	230 (30.8)	< 0.001
AKI within 2 days	862 (69.5)	937 (75.5)	0.001
AKI within 7 days	959 (77.3)	1008 (81.2)	0.018
Ventilation-free day in 28 days	20.79 (9.86)	21.22 (9.80)	0.272
Vasopressor-free day in 28 days*	16.95 (13.31)	21.15 (11.00)	< 0.001
IVF of day1 (mean (SD))	3100.88 (3163.26)	4167.19 (3838.39)	< 0.001
IVF of day2 (mean (SD))	2274.16 (2405.90)	2058.66 (2357.80)	0.045
IVF of day3 (mean (SD))	2178.88 (2492.15)	1647.62 (2097.16)	< 0.001

*CVP* central venous pressure, *AKI* acute kidney injury, *IVF* intravenous fluid

^*^data from MIMIC-III database only

## Discussion

The goal of current study was to investigate the application value of CVP in patients with severe coma in the ICU. We found that early CVP was independently associated with lower all-cause in-hospital mortality in severe coma patients (GCS score 3–8). We also found the association of the initial CVP time and in-hospital mortality was presented as a “U-shape” in the RCS curve. As the initial CVP time increases, the risk of all-cause in-hospital mortality increases.

Because of the standardization of advances in technologies, such as MVs, in ICU management, more and more comatose patients are surviving life-threatening diseases. However, the high mortality rate and cost for this type of patient are still among the most severe clinical and social problems [23, 24]. Any efforts that could increase the chances of reasonable functional outcomes in patients with coma are of utmost clinical and ethical significance [25].

This is the first study to investigate the association between the early CVP and clinical outcomes in patients with severe coma. The current study was a post hoc analysis. The data included in this study were from the eICU and MIMIC-III databases, which contain ICU patients across more than 200 different hospitals in the USA, with varying CVP practices. Our analysis leverages the availability of time-stamped demographic information, comorbidities, vital signs, laboratory results, etc., to investigate whether CVP could benefit patients with severe coma. The results of the study were highly consistent across different sensitivity analyses.

Conflicting evidence on the application of the CVP has been reported in previous studies. Eskesen et al. found that the application value of the CVP measurement is low in a re-analysis of 1148 patient data sets [5]. They reported that only in certain extreme cases, such as higher or lower, does CVP measurement demonstrate some of its value in liquid management. Marik et al. conducted a meta-analysis and reported that only 57% of patients were fluid responders, and the correlation between CVP level and stroke volume index was not strong; thus, the use of the CVP for fluid resuscitation should be abandoned in widespread practice due to insufficient evidence. However, many studies have reported the association between the CVP levels and patients’ clinical outcomes with various conditions. For example, Chen et al. found in a meta-analysis that a higher CVP value was associated with higher mortality (odds ratio [OR] 1.65) and a greater risk of AKI (OR, 2.09). Quail et al. found that a higher CVP is associated with a higher risk of early Fontan failure after total cavopulmonary connections [10]. Liu et al. also found that a controlled lower CVP could reduce blood loss during hepatectomy and recommended the promotion of CVP use in clinical settings [12].

Despite the conflicting evidence reported and the value of the CVP being questioned, some clinicians still recommend the use of the CVP in clinical settings [3, 7]. One recommendation is to use CVP as a stop symbol in liquid management. Weil and Henning first proposed this approach and suggested that fluid administration could be stop as the CVP increased by 5 cmH2O or more [26]. However, Hamzaoui et al. reported that changes in the stroke volume could not be reflected by the CVP levels. Therefore, the CVP levels could not represent as a criterion for predicting fluid responsiveness [6].

Whatever the approach to CVP application, some evidence also indicates that CVP use is associated with a better clinical outcome in some patients [14, 15]. Chen et al. reported the association between the CVP using and lower 28-day mortality in septic patients [14]. Tang et al. reported that the CVP could improve the clinical outcomes of patients with acute respiratory distress syndrome [15]. However, both studies were from a single-center database; thus, the generalization of the results could be hindered. In these multicenter studies, we found that severe comas patients with CVP suing have a lower in-hospital mortality than those without CVP. Moreover, we found that with the delay in the initial CVP time, the risk of in-hospital mortality is increasing. In the secondary outcomes, we found that CVP was also associated with ICU mortality, 28-day mortality, 365-day mortality, and more days without vasopressor drugs. We believe that these results confirm the application value of CVP in comatose patients.

The detailed influence of the CVP on therapeutic interventions was difficult to explore due to the retrospective design. Previous studies reported that the trigger caused by CVP, such as fluid therapy, could lower lactate levels of patients, thus could improve outcomes [14, 15]. Semler et al. found that different fluid management based on initial CVP values could influence outcomes of acute respiratory distress syndrome patients, and conservative fluid administration could lower the mortality of patients with a low initial CVP [27]. Wang et al. found that patients who have a peak CVP value more than 12 mmHg also have worse organ function, higher SOFA score and longer hospital stay [2]. These results indicate that CVP could positively impact the latter management of patients. In the current study, we found that the IVF was significantly different between patients with and -out CVP. Generally, IVF volumes in patients with CVP were higher on day 1 and lower on day 3 compared to those in the non-CVP group. Most patients (62.1%) with CVP had an initial CVP value of ≤ 12 mmH2O. It is difficult to investigate whether the difference in the IVF on the first day was influenced by the CVP. However, relatively free fluid management may be safe when the levels maintaining as low as possible [3]. Fluid management is a double-edged sword. Correction of intravascular hypovolemia is critical for preventing and managing AKI, but excessive fluid administration could also increases the risk of AKI [28, 29]. In the current study, we also discovered that the 2-day and 7-day AKI was higher in patients with CVP, which is different from that in previous studies. The causal relationship between the increased risk of AKI and the CVP requires further research and evaluation.

### Limitations

Our study had some limitations. First, although the GCS has been used for more than 40 years and is the most widespread measurement in brain injury studies [20], using the GCS score of 3–8 to define severe coma in patients in the ICU could still introduce patient selection bias. Meanwhile, all data in this study were extracted using a structured query language, which may also cause misclassification of patients. Second, considering that the significant covariate imbalance between the CVP and non-CVP groups may influence the explanation of the results, we used a PSM approach to minimize the differences and performed several sensitivity analyses. However, covariate imbalance was still observed for some variables. Third, although we included patients from two large databases from more than 200 hospitals to improve the generalization of the results, the retrospective design may still introduce some analysis bias. Fourth, because CVP values are generally measured and read manually, measurement and reading errors cannot be avoided. Finally, CVP-related management changes are complex in clinical practice. The nature of post hoc analysis of the current study should not be ignored. Although we found a robust association between CVP and the primary outcome, the causal relationship of this condition remains unclear and should be investigated in further studies.

## Conclusion

In critically ill patients with severe coma (GCS score 3–8), early CVP measurements is independently associated with lower in-hospital mortality. Among patients with CVP measurements, in-hospital mortality increased with a delayed initial CVP measurement.

## Supplementary Information

**Additional file 1: Table S1.** Missing value for included variables in this study. **Table S2.** Full multivariate model assessing the impact of central venous pressure on in hospital mortality in the original cohort. **Table S3.** Full multivariate model assessing the impact of central venous pressure on in hospital mortality in the PSM cohort. **Table S4.** Full multivariate model assessing the impact of central venous pressure on in hospital mortality in the IPTW cohort. **Table S5.** Full multivariate model assessing the impact of central venous pressure on in hospital mortality in the sIPTW cohort.

## Data Availability

MIMIC III and eICU-CRD databases are available to researchers through credentialed access on the PhysioNet.

## Abbreviations

AKI: Acute kidney injury
CVP: Central venous pressure
GCS: Glasgow Coma Scale
HR: Hazard ratio (HR)
eICU-CRD/eICU: ICU Collaborative Research Database
IVF: Intravenous fluid
IPTW: Inverse probability treatment weighting
MIMIC-III: Medical Information Mart for Intensive Care III
OR: Odds ratio
PSM: Propensity score matching
RCS: Restricted cubic splines
sIPTW: Stabilized IPTW
SD: Standard deviation
