# Supplementary material for: Association between central venous pressure measurement and outcomes in critically ill patients with severe coma
Source: Eur J Med Res. 2023 Jan 18;28:35. doi: 10.1186/s40001-022-00981-9 (PMC9847142; doi:10.1186/s40001-022-00981-9)
Supplement: Supplementary file 1 — Additional file 1: Table S1. Missing value for included variables in this study. Table S2. Full multivariate model assessing the impact of central venous pressure on in hospital mortality in the original cohort. Table S3. Full multivariate model assessing the impact of central venous pressure on in hospital mortality in the PSM cohort. Table S4. Full multivariate model assessing the impact of central venous pressure on in hospital mortality in the IPTW cohort. Table S5. Full multivariate model assessing the impact of central venous pressure on in hospital mortality in the sIPTW cohort. [file 40001_2022_981_MOESM1_ESM.docx]

**Additional file information**

Table S1 Missing value for included variables in this study.

| variable | Missing (number) | Percent missing (%) |
| --- | --- | --- |
| Lactate | 2652 | 35.90577 |
| day3 intravenous fluid | 2578 | 34.90387 |
| day2 intravenous fluid | 2009 | 27.20011 |
| day1 intravenous fluid | 1053 | 14.2567 |
| Bicarbonate | 527 | 7.135121 |
| MAP | 452 | 6.119686 |
| Platelet | 299 | 4.048199 |
| WBC | 285 | 3.858652 |
| Hemoglobin | 279 | 3.777417 |
| Chloride | 254 | 3.438939 |
| BUN | 244 | 3.303547 |
| Temperature | 225 | 3.046304 |
| Sodium | 216 | 2.924452 |
| Potassium | 215 | 2.910913 |
| HR | 144 | 1.949634 |
| Ethnicity | 58 | 0.785269 |

Table S2 Full multivariate model assessing the impact of central venous pressure on in hospital mortality in the original cohort.

|  | Hazard Ratio | 2.5% | 97.5% | P value |
| --- | --- | --- | --- | --- |
| Primary diagnosis |  |  |  |  |
| Circulatory disease |  |  |  |  |
| Digestive disease | 0.4742 | 0.34024 | 0.6608 | 1.05E-05 |
| Endocrine disease | 0.6912 | 0.52727 | 0.906 | 0.007486 |
| Infectious disease | 0.7686 | 0.63788 | 0.9261 | 0.005661 |
| Injury disease | 0.7914 | 0.6648 | 0.9421 | 0.008538 |
| Mental disease | 0.2923 | 0.09358 | 0.9129 | 0.03427 |
| Neoplasm disease | 0.4115 | 0.26901 | 0.6295 | 4.25E-05 |
| Nervous disease | 0.5778 | 0.4537 | 0.7359 | 8.81E-06 |
| Other disease | 0.7774 | 0.64544 | 0.9362 | 0.007941 |
| Respiratory disease | 0.8328 | 0.69494 | 0.9981 | 0.047584 |
| Urinary disease | 0.6642 | 0.40562 | 1.0875 | 0.103824 |
| GCS | 0.8406 | 0.81772 | 0.8641 | < 2e-16 |
| CVP use | 0.6263 | 0.55311 | 0.7091 | 1.52E-13 |
| Gender1 |  |  |  |  |
| male |  |  |  |  |
| female | 0.9057 | 0.81964 | 1.0008 | 0.05187 |
| Age | 1.0138 | 1.01041 | 1.0172 | 8.47E-16 |
| Ethnicity |  |  |  |  |
| White |  |  |  |  |
| Black | 0.7582 | 0.65184 | 0.8819 | 3.32E-04 |
| Asian | 0.6071 | 0.3914 | 0.9418 | 0.025902 |
| Hispanic | 0.6322 | 0.47323 | 0.8446 | 0.001915 |
| Other | 1.1641 | 0.98587 | 1.3746 | 0.073112 |
| Unit |  |  |  |  |
| CICU |  |  |  |  |
| MICU | 1.3379 | 1.13503 | 1.5769 | 0.000521 |
| MSICU | 1.6813 | 1.46103 | 1.9349 | 4.15E-13 |
| NICU | 2.1476 | 1.70531 | 2.7046 | 8.22E-11 |
| SICU | 1.2581 | 1.04031 | 1.5214 | 0.017909 |
| CHF | 0.9495 | 0.80607 | 1.1184 | 0.535002 |
| AF | 0.8246 | 0.70602 | 0.9632 | 0.014975 |
| Renal | 1.0057 | 0.85252 | 1.1863 | 0.946526 |
| Liver | 0.7982 | 0.60804 | 1.0479 | 0.104563 |
| COPD | 1.0737 | 0.87987 | 1.3101 | 0.484025 |
| CAD | 0.4445 | 0.35387 | 0.5585 | 3.27E-12 |
| Stroke | 1.776 | 1.51525 | 2.0817 | 1.35E-12 |
| MT | 0.8737 | 0.71558 | 1.0668 | 0.185077 |
| HR | 1.0006 | 0.99851 | 1.0027 | 0.582878 |
| MAP | 0.9994 | 0.9974 | 1.0015 | 0.590415 |
| Temperature | 0.9694 | 0.94633 | 0.993 | 1.14E-02 |
| WBC | 1.0045 | 1.0012 | 1.0077 | 0.007283 |
| Hemoglobin | 0.9968 | 0.97777 | 1.0162 | 0.742428 |
| Platelet | 1.0005 | 1.00001 | 1.0009 | 0.04453 |
| Potassium | 0.9307 | 0.88064 | 0.9836 | 0.010892 |
| Bicarbonate | 0.9813 | 0.97182 | 0.9908 | 0.000126 |
| Chloride | 0.9904 | 0.98421 | 0.9966 | 2.52E-03 |
| BUN | 1.0029 | 1.00077 | 1.005 | 0.007418 |
| Lactate | 1.0813 | 1.0679 | 1.0949 | < 2e-16 |
| Sedative use | 0.582 | 0.52146 | 0.6496 | < 2e-16 |
| Vasopressor use | 1.1382 | 1.01445 | 1.2769 | 0.027494 |
| MV use | 1.6413 | 1.41209 | 1.9077 | 1.07E-10 |
| SOFA | 1.0861 | 1.06784 | 1.1047 | < 2e-16 |
| days in ICU | 0.8327 | 0.81995 | 0.8456 | < 2e-16 |

CVP, central venous pressure; GCS, Glasgow Coma Scale; CCI, Charlson Comorbidity Index; TBI, traumatic brain injuries; CHF, Congestive heart failure; AF, Atrial fibrillation; COPD, Chronic obstructive pulmonary disease; CAD, Coronary artery disease; HR, Heart rate; MAP, Mean arterial pressure; WBC, white blood cell; BUN, blood urea nitrogen; MV, mechanical ventilation; SOFA, sequential organ failure assessment.

Table S3 Full multivariate model assessing the impact of central venous pressure on in hospital mortality in the PSM cohort.

|  | Hazard Ratio | 2.5% | 97.5% | P value |
| --- | --- | --- | --- | --- |
| Primary diagnosis |  |  |  |  |
| Circulatory disease | ref |  |  |  |
| Digestive disease | 0.5415 | 0.36593 | 0.8013 | 0.002157 |
| Endocrine disease | 0.7231 | 0.51181 | 1.0218 | 0.066077 |
| Infectious disease | 0.7999 | 0.64033 | 0.9992 | 0.049229 |
| Injury disease | 0.8147 | 0.64128 | 1.0351 | 0.093451 |
| Mental disease | 0.3437 | 0.08485 | 1.392 | 0.134506 |
| Neoplasm disease | 0.3172 | 0.1858 | 0.5415 | 2.58E-05 |
| Nervous disease | 0.6083 | 0.39175 | 0.9447 | 0.02687 |
| Other disease | 0.7381 | 0.57466 | 0.9479 | 0.017363 |
| Respiratory disease | 0.9215 | 0.70925 | 1.1973 | 0.540622 |
| Urinary disease | 0.3734 | 0.15182 | 0.9186 | 0.031958 |
| GCS | 0.8659 | 0.83593 | 0.8968 | 1.00E-15 |
| CVP use | 0.5791 | 0.50953 | 0.6581 | < 2e-16 |
| Gender |  |  |  |  |
| Male | ref |  |  |  |
| Female | 1.067 | 0.93592 | 1.2164 | 0.332207 |
| Age | 1.0199 | 1.01525 | 1.0246 | < 2e-16 |
| Ethnicity |  |  |  |  |
| White | ref |  |  |  |
| Black | 0.8341 | 0.67592 | 1.0293 | 0.09095 |
| Asian | 0.6625 | 0.39243 | 1.1185 | 0.123336 |
| Hispanic | 0.7933 | 0.54544 | 1.1538 | 0.22577 |
| Other | 1.2652 | 1.03967 | 1.5397 | 0.018861 |
| Unit |  |  |  |  |
| CICU | ref |  |  |  |
| MICU | 1.6146 | 1.32729 | 1.9642 | 1.65E-06 |
| MSICU | 1.8546 | 1.56348 | 2.1998 | 1.34E-12 |
| NICU | 2.2871 | 1.60835 | 3.2523 | 4.12E-06 |
| SICU | 1.1575 | 0.90583 | 1.4792 | 0.242266 |
| CHF | 0.9547 | 0.79074 | 1.1526 | 0.629605 |
| AF | 0.6965 | 0.5784 | 0.8388 | 0.000137 |
| Renal | 1.0303 | 0.84013 | 1.2636 | 0.774211 |
| Liver | 0.799 | 0.59519 | 1.0727 | 0.135409 |
| COPD | 1.1038 | 0.88767 | 1.3726 | 0.374372 |
| CAD | 0.4567 | 0.35613 | 0.5856 | 6.56E-10 |
| Stroke | 2.1929 | 1.75173 | 2.7453 | 7.32E-12 |
| MT | 1.1273 | 0.89084 | 1.4266 | 0.318421 |
| HR | 0.9992 | 0.99648 | 1.0019 | 0.558746 |
| MAP | 0.9992 | 0.99619 | 1.0022 | 0.594593 |
| Temperature | 0.9632 | 0.93088 | 0.9966 | 0.031247 |
| WBC | 1.0042 | 1.00072 | 1.0077 | 0.018001 |
| Hemoglobin | 1.0324 | 1.00598 | 1.0596 | 0.015936 |
| Platelet | 1.0007 | 1.00008 | 1.0013 | 0.027195 |
| Potassium | 0.9503 | 0.88755 | 1.0176 | 0.144204 |
| Bicarbonate | 0.9849 | 0.97271 | 0.9973 | 0.01713 |
| Chloride | 1.0877 | 1.06928 | 1.1065 | < 2e-16 |
| BUN | 0.9853 | 0.97702 | 0.9936 | 0.000542 |
| Lactate | 1.0038 | 1.00077 | 1.0067 | 0.013481 |
| Sedative use | 0.5405 | 0.46713 | 0.6254 | < 2e-16 |
| Vasopressor use | 1.0558 | 0.91413 | 1.2193 | 0.460298 |
| MV use | 1.6561 | 1.35841 | 2.0189 | 6.03E-07 |
| SOFA | 1.0934 | 1.06969 | 1.1177 | 1.50E-15 |
| days in ICU | 0.8511 | 0.83658 | 0.8659 | < 2e-16 |

CVP, central venous pressure; GCS, Glasgow Coma Scale; CCI, Charlson Comorbidity Index; TBI, traumatic brain injuries; CHF, Congestive heart failure; AF, Atrial fibrillation; COPD, Chronic obstructive pulmonary disease; CAD, Coronary artery disease; HR, Heart rate; MAP, Mean arterial pressure; WBC, white blood cell; BUN, blood urea nitrogen; MV, mechanical ventilation; SOFA, sequential organ failure assessment.

Table S4 Full multivariate model assessing the impact of central venous pressure on in hospital mortality in the IPTW cohort.

|  | Hazard Ratio | 2.5% | 97.5% | P value |
| --- | --- | --- | --- | --- |
| Primary diagnosis |  |  |  |  |
| Circulatory disease | ref |  |  |  |
| Digestive disease | 0.5233 | 0.32705 | 0.8373 | 0.006926 |
| Endocrine disease | 0.7657 | 0.55713 | 1.0525 | 0.099996 |
| Infectious disease | 0.8476 | 0.67903 | 1.0581 | 0.14406 |
| Injury disease | 0.7702 | 0.60995 | 0.9724 | 0.028179 |
| Mental disease | 0.2551 | 0.09191 | 0.7078 | 0.008705 |
| Neoplasm disease | 0.4841 | 0.3064 | 0.765 | 0.001885 |
| Nervous disease | 0.5619 | 0.39097 | 0.8075 | 1.83E-03 |
| Other disease | 0.7586 | 0.6022 | 0.9555 | 0.018953 |
| Respiratory disease | 0.9831 | 0.7145 | 1.3525 | 0.916373 |
| Urinary disease | 0.5643 | 0.30872 | 1.0315 | 0.063023 |
| GCS | 0.8233 | 0.79623 | 0.8514 | < 2e-16 |
| CVP use | 0.6363 | 0.54891 | 0.7375 | 1.97E-09 |
| Gender |  |  |  |  |
| Male | ref |  |  |  |
| Female | 0.8814 | 0.7769 | 1 | 0.049946 |
| Age | 1.0135 | 1.009 | 1.018 | 3.18E-09 |
| Ethnicity |  |  |  |  |
| White | ref |  |  |  |
| Black | 0.7575 | 0.62686 | 0.9154 | 4.04E-03 |
| Asian | 0.632 | 0.4063 | 0.9831 | 0.041797 |
| Hispanic | 0.6647 | 0.47949 | 0.9214 | 0.014245 |
| Other | 1.1227 | 0.87835 | 1.4349 | 0.355472 |
| Unit |  |  |  |  |
| CICU | ref |  |  |  |
| MICU | 1.2137 | 1.00431 | 1.4668 | 0.045018 |
| MSICU | 1.5778 | 1.34606 | 1.8495 | 1.84E-08 |
| NICU | 2.2308 | 1.66371 | 2.9911 | 8.24E-08 |
| SICU | 1.1488 | 0.88797 | 1.4863 | 2.91E-01 |
| CHF | 0.8649 | 0.6886 | 1.0864 | 0.212209 |
| AF | 0.8782 | 0.69188 | 1.1147 | 0.285735 |
| Renal | 0.9245 | 0.71863 | 1.1893 | 0.541231 |
| Liver | 0.7892 | 0.57818 | 1.0772 | 0.135784 |
| COPD | 0.9752 | 0.76226 | 1.2477 | 0.841818 |
| CAD | 0.7519 | 0.52211 | 1.0828 | 0.12536 |
| Stroke | 1.7337 | 1.4393 | 2.0884 | 6.83E-09 |
| MT | 0.8545 | 0.65048 | 1.1225 | 2.59E-01 |
| HR | 1.0003 | 0.99768 | 1.003 | 0.816747 |
| MAP | 0.9994 | 0.9969 | 1.002 | 0.660997 |
| Temperature | 0.9507 | 0.91032 | 0.9929 | 0.022627 |
| WBC | 1.0045 | 1.00289 | 1.006 | 2.47E-08 |
| Hemoglobin | 1.0012 | 0.97588 | 1.0271 | 9.29E-01 |
| Platelet | 1.0005 | 0.99996 | 1.0011 | 0.067952 |
| Potassium | 0.9692 | 0.8975 | 1.0467 | 0.4255 |
| Bicarbonate | 0.9744 | 0.95989 | 0.989 | 0.000664 |
| Chloride | 0.9907 | 0.98368 | 0.9978 | 9.91E-03 |
| BUN | 1.0025 | 0.99957 | 1.0054 | 9.40E-02 |
| Lactate | 1.063 | 1.04127 | 1.0853 | 6.99E-09 |
| Sedative use | 0.6036 | 0.52927 | 0.6885 | 5.30E-14 |
| Vasopressor use | 1.1702 | 1.01906 | 1.3437 | 2.59E-02 |
| MV use | 1.5461 | 1.28603 | 1.8588 | 3.53E-06 |
| SOFA | 1.0905 | 1.06788 | 1.1135 | 4.88E-16 |
| days in ICU | 0.8515 | 0.79492 | 0.9121 | 4.62E-06 |

CVP, central venous pressure; GCS, Glasgow Coma Scale; CCI, Charlson Comorbidity Index; TBI, traumatic brain injuries; CHF, Congestive heart failure; AF, Atrial fibrillation; COPD, Chronic obstructive pulmonary disease; CAD, Coronary artery disease; HR, Heart rate; MAP, Mean arterial pressure; WBC, white blood cell; BUN, blood urea nitrogen; MV, mechanical ventilation; SOFA, sequential organ failure assessment.

Table S5 Full multivariate model assessing the impact of central venous pressure on in hospital mortality in the sIPTW cohort.

|  | Hazard Ratio | 2.5% | 97.5% | P value |
| --- | --- | --- | --- | --- |
| Primary diagnosis |  |  |  |  |
| Circulatory disease | ref |  |  |  |
| Digestive disease | 0.5544 | 0.3588 | 0.8567 | 0.007896 |
| Endocrine disease | 0.7393 | 0.52464 | 1.0418 | 0.08436 |
| Infectious disease | 0.8347 | 0.65107 | 1.07 | 0.153847 |
| Injury disease | 0.7333 | 0.56177 | 0.9572 | 0.022496 |
| Mental disease | 0.278 | 0.08952 | 0.8636 | 0.026854 |
| Neoplasm disease | 0.4984 | 0.28893 | 0.8596 | 0.012288 |
| Nervous disease | 0.5732 | 0.35788 | 0.9182 | 0.02061 |
| Other disease | 0.7448 | 0.56897 | 0.975 | 0.032034 |
| Respiratory disease | 1.014 | 0.72391 | 1.4204 | 0.935512 |
| Urinary disease | 0.4564 | 0.21181 | 0.9835 | 0.045232 |
| GCS | 0.8264 | 0.79439 | 0.8596 | < 2e-16 |
| CVP use | 0.649 | 0.56203 | 0.7495 | 3.93E-09 |
| Gender1 |  |  |  |  |
| Male | ref |  |  |  |
| Female | 0.8662 | 0.74784 | 1.0033 | 0.055318 |
| Age | 1.0127 | 1.00762 | 1.0177 | 7.64E-07 |
| Ethnicity |  |  |  |  |
| White | ref |  |  |  |
| Black | 0.7459 | 0.5999 | 0.9275 | 0.008358 |
| Asian | 0.6704 | 0.40405 | 1.1123 | 0.121601 |
| Hispanic | 0.6884 | 0.48217 | 0.9829 | 0.039886 |
| Other | 1.1179 | 0.87162 | 1.4336 | 0.380168 |
| Unit |  |  |  |  |
| CICU | ref |  |  |  |
| MICU | 1.311 | 1.0562 | 1.6272 | 0.014053 |
| MSICU | 1.7215 | 1.44848 | 2.0461 | 7.06E-10 |
| NICU | 2.4828 | 1.76015 | 3.5021 | 2.20E-07 |
| SICU | 1.3131 | 1.01334 | 1.7015 | 3.94E-02 |
| CHF | 0.8709 | 0.69425 | 1.0925 | 0.231996 |
| AF | 0.8312 | 0.65089 | 1.0615 | 0.13841 |
| Renal | 0.911 | 0.71589 | 1.1592 | 0.448157 |
| Liver | 0.7333 | 0.53824 | 0.9991 | 0.049326 |
| COPD | 1.1664 | 0.9041 | 1.5048 | 0.236287 |
| CAD | 0.6138 | 0.43247 | 0.8711 | 0.006289 |
| Stroke | 1.7317 | 1.4095 | 2.1276 | 1.72E-07 |
| MT | 0.8739 | 0.64076 | 1.1919 | 3.95E-01 |
| HR | 0.9999 | 0.99671 | 1.0031 | 0.961744 |
| MAP | 0.9999 | 0.99706 | 1.0027 | 0.923658 |
| Temperature | 0.9399 | 0.89881 | 0.983 | 0.006674 |
| WBC | 1.0045 | 1.00264 | 1.0063 | 1.79E-06 |
| Hemoglobin | 1.0107 | 0.98095 | 1.0414 | 4.84E-01 |
| Platelet | 1.0007 | 0.99999 | 1.0014 | 0.052869 |
| Potassium | 0.9531 | 0.87803 | 1.0347 | 0.251857 |
| Bicarbonate | 0.9722 | 0.95426 | 0.9905 | 0.003029 |
| Chloride | 0.9908 | 0.9824 | 0.9992 | 3.14E-02 |
| BUN | 1.0024 | 0.99908 | 1.0057 | 1.56E-01 |
| Lactate | 1.0533 | 1.0258 | 1.0815 | 0.000119 |
| Sedative use | 0.6067 | 0.52107 | 0.7065 | 1.25E-10 |
| Vasopressor use | 1.1407 | 0.97231 | 1.3383 | 1.06E-01 |
| MV use | 1.4094 | 1.14357 | 1.737 | 0.00129 |
| SOFA | 1.1001 | 1.07419 | 1.1267 | 4.59E-15 |
| days in ICU | 0.8479 | 0.79932 | 0.8995 | 4.32E-08 |

CVP, central venous pressure; GCS, Glasgow Coma Scale; CCI, Charlson Comorbidity Index; TBI, traumatic brain injuries; CHF, Congestive heart failure; AF, Atrial fibrillation; COPD, Chronic obstructive pulmonary disease; CAD, Coronary artery disease; HR, Heart rate; MAP, Mean arterial pressure; WBC, white blood cell; BUN, blood urea nitrogen; MV, mechanical ventilation; SOFA, sequential organ failure assessment.
